# Supplementary material for: wMel Wolbachia alters female post-mating behaviors and physiology in the dengue vector mosquito Aedes aegypti
Source: Commun Biol. 2023 Aug 21;6:865. doi: 10.1038/s42003-023-05180-8 (PMC10442437; doi:10.1038/s42003-023-05180-8)
Supplement: Supplementary file 2 — Supplementary Information [file 42003_2023_5180_MOESM2_ESM.pdf]

wMel *Wolbachia* alters female post-mating behaviors and physiology in the dengue vector mosquito *Aedes aegypti*

Jessica Osorio[1], Sara Villa-Arias[1,2], Carolina Camargo[3], Luis Felipe Ramírez-Sánchez[1], Luisa María Barrientos[1], Carolina Bedoya[1], Guillermo Rúa-Uribe[4], Steve Dorus[5], Catalina Alfonso-Parra[1,2*], Frank W. Avila[1*]

1. Max Planck Tandem Group in Mosquito Reproductive Biology, Universidad de Antioquia, Medellín, Colombia
2. Instituto Colombiano de Medicina Tropical, Universidad CES, Sabaneta, Colombia
3. Centro de Investigación de la caña de azúcar CENICAÑA, Valle del Cauca, Colombia
4. Grupo Entomología Médica, Universidad de Antioquia, Medellín, Colombia
5. Center for Reproductive Evolution, Syracuse University, Syracuse, USA

***Corresponding authors:**

Frank W. Avila: grupotandem.mosquito@udea.edu.co

Catalina Alfonso-Parra: catalfonso@gmail.com

Supplemental Tables and Figures

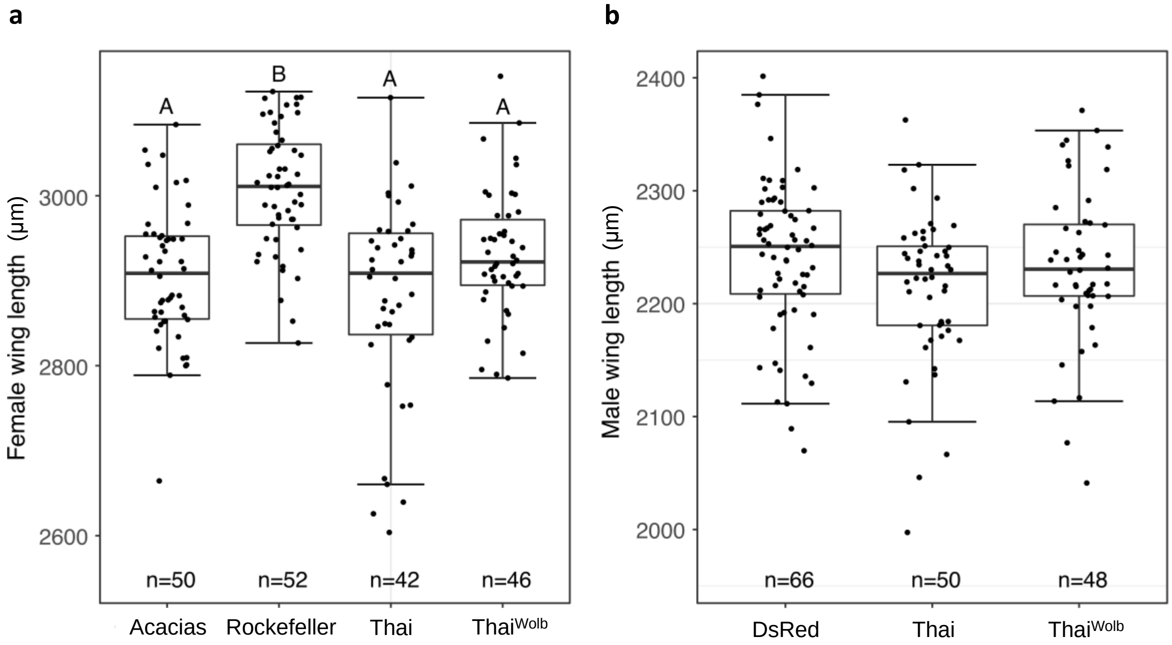

Supplementary Figure 1. Wing lengths of female (**a**) and male (**b**) mosquitoes used in our assays. Groups denoted by different letters are significantly different for a post hoc Tukey test (p < 0.05). Females displayed significant differences in sizes (Df = 3, F = 19.83, p = 3.39e-11), with Rockefeller females significantly larger than Thai, Thai^{Wolb}, and Acacias females. No differences were observed in sizes between Thai^{Wolb} and Thai males or females (females: p = 0.1; males: p = 0.33). For the box plots, the middle horizontal line represents the median, the lower and upper margins of the box represent the 25th and 75th quartiles, and the whiskers extend to the minimum and maximum of the data (excluding outliers, shown as points outside the whiskers).

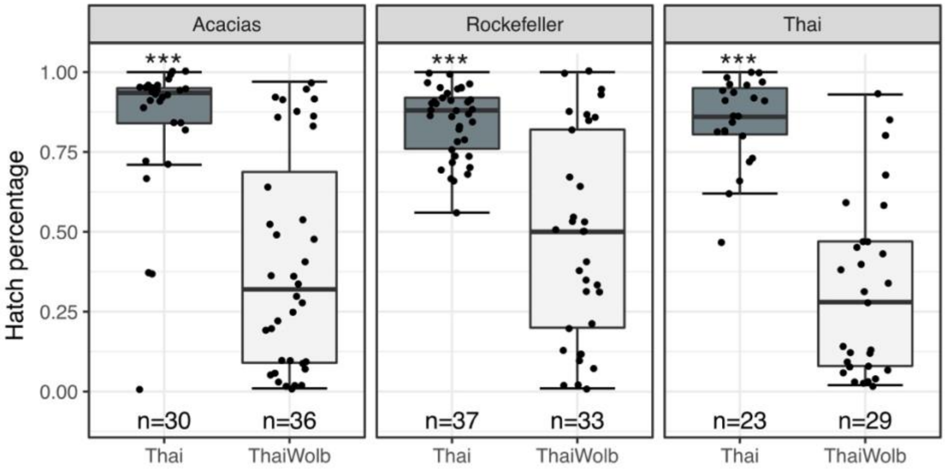

Supplementary Figure 2. Fertility of multiply mated Acacias, Rockefeller and Thai strain females initially mated to a Thai^{Wolb} male and re-inseminated by an uninfected DsRed male. For the box plots, the middle horizontal line represents the median, the lower and upper margins of the box represent the 25th and 75th quartiles, and the whiskers extend to the minimum and maximum of the data (excluding outliers, shown as points outside the whiskers). *** $p < 0.001$.

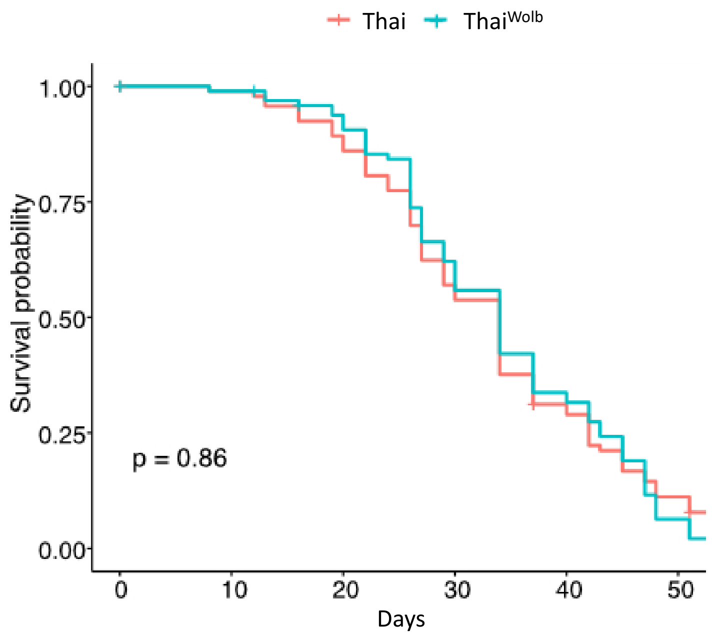

Supplementary Figure 3. *Wolbachia* infection does not impact longevity of *Aedes aegypti* males. Longevity of Thai and Thai^{Wolb} males (N_{Thai} = 91; $N_{ThaiWolb}$ = 95).

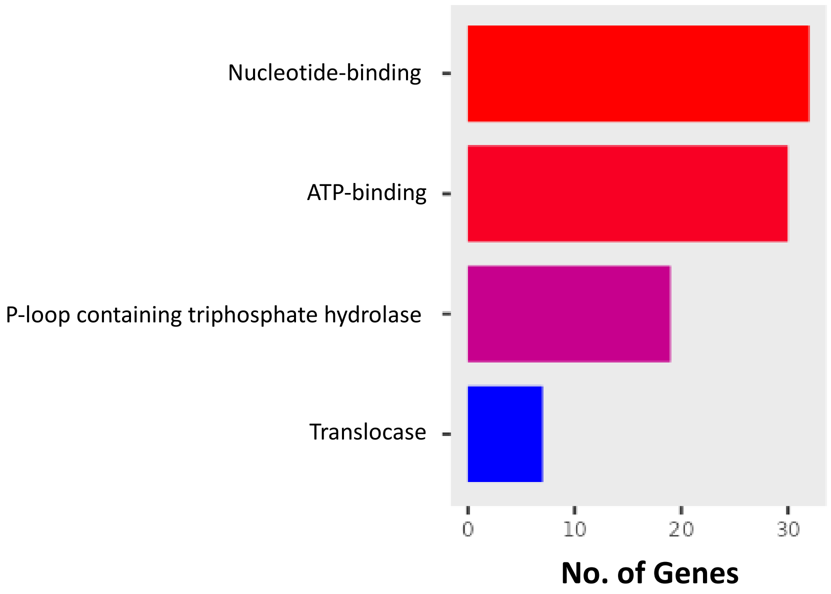

Supplementary Figure 4. Enrichment of Gene Ontology (GO) terms of *Wolbachia* genes that code for proteins transferred in the ejaculate during mating by Thai^{Wolb} males.

	Female	1st mating male	% re-copulated	N
**24 hours**	Thai	Thai	0	25
		$Thai^{Wolb}$	0	25
**7 days**	$Thai^{Wolb}$	Thai	0.04%	45
		$Thai^{Wolb}$	0	45

Supplementary Table 1. Re-mating incidence of Thai females 24 h and 7 d after an initial mating to the indicated male.

Seminal proteins identified only in Thai^{Wolb} male ejaculates		
**Accession**	**VectorBase ID**	**Description**
XP_001649118.1	AAEL004448	torsin-like protein
XP_021704077.1	AAEL007911	dynein heavy chain 2
XP_001657568.1	AAEL006196	adipocyte plasma membrane-associated protein
XP_001660989.2	AAEL010673	NADH dehydrogenase, putative
XP_021708222.1	AAEL018354	oxysterol-binding protein 1
XP_001652605.1	AAEL007184	V-type proton ATPase subunit G
XP_001653970.1	AAEL001779	bax inhibitor 1
XP_021707235.1	AAEL019855	serine/threonine-protein kinase
XP_001659317.1	AAEL001452	Unspecified product
XP_021699872.1	AAEL011280	voltage-dependent p/q type calcium channel
XP_001653703.2	AAEL009158	unspecified product
XP_021694162.1	AAEL020476	unspecified product

XP_021704494.1	AAEL006028	unspecified product
**Seminal proteins identified only in Thai male ejaculates**		
**Accession**	**VectorBase ID**	**Description**
XP_021703552.1	AAEL024503	unspecified product

Supplementary Table 2. Seminal proteins identified only in the ejaculate of *Wolbachia* infected males (top) or in the ejaculates of control males (bottom). Only proteins that had a PSM ≥ 5 were considered in this analysis.

CI-phenotype associated proteins		
**Accession**	**Gene**	**Protein**
RPOBC_WOLPM	WD0024	Bifunctional DNA-directed RNA polymerase subunit beta-beta
Q73HV7_WOLPM	WD0433	propionyl-CoA carboxylase
Q73HT1_WOLPM	WD0462	Uncharacterized protein
Q73HH8_WOLPM	WD0582	Regulatory protein RepA, putative
Q73HF5_WOLPM	WD0609	Regulatory protein RepA, putative
Q73GJ7_WOLPM	WD0954	Aspartate-semialdehyde dehydrogenase
Q73FY5_WOLPM	WD1187	Conserved domain protein
**WO phage proteins**		
**Accession**	**Gene**	**Protein**
Q73HN8_WOLPM	WD0511	Rpn family recombination-promoting nuclease/putative transposase
Q73HN6_WOLPM	WD0513	TcdB_toxin_midN domain-containing protein
Q73HI3_WOLPM	WD0577	Phage related protein
Q73HH8_WOLPM	WD0582	Regulatory protein RepA, putative

Q73HH7_WOLPM	WD0583	AAA domain-containing protein
**Q73HF5_WOLPM**	**WD0609**	**Regulatory protein RepA, putative**
**Q73HF4_WOLPM**	**WD0610**	**Helicase, SNF2 family**
**Q73HE9_WOLPM**	**WD0615**	**Conserved domain protein**
**Q73HE8_WOLPM**	**WD0616**	**ABC transporter, permease/ATP-binding protein, putative**
**Q73HD6_WOLPM**	**WD0630**	**PH domain-containing protein**
**M1FTP9_WOLPM**	**WD0633**	**Ankyrin domain protein**

Supplementary Table 3. Paternally transferred *Wolbachia* proteins that modify CI-phenotypes[74] and/or that are part of the phage WO sequences or WO-like Islands[76]. Phage WO proteins that are associated with the Eukaryotic Association Module[77] are shown in **bold**.
