# Supplementary material for: wMel Wolbachia alters female post-mating behaviors and physiology in the dengue vector mosquito Aedes aegypti
Source: Commun Biol. 2023 Aug 21;6:865. doi: 10.1038/s42003-023-05180-8 (PMC10442437; doi:10.1038/s42003-023-05180-8)
Supplement: Supplementary file 3 — Description of Additional Supplementary Files [file 42003_2023_5180_MOESM3_ESM.pdf]

Description of Additional Supplementary Files

File name: Supplementary Data 1

Description: Mass spectrometry data of the paternally transferred Wolbachia proteins identified.

File name: Supplementary Data 2

Description: Raw data used for the generation of Figures 1, 2, and 3.
